# Association between maternal employment status and presence of children with major congenital anomalies in Denmark

**DOI:** 10.1186/s12889-024-18190-w

**Published:** 2024-03-06

**Authors:** Kyung Mi Kim, Dóra Körmendiné Farkas, Venus Wong, Cathrine Fonnesbech Hjorth, Erzsébet Horváth-Puhó, Eli Cahan, Eyal Cohen, Nirav R. Shah, Henrik Toft Sørensen, Arnold Milstein

**Affiliations:** 1 Clinical Excellence Research Center, School of Medicine, Stanford University, Stanford, CA USA; 2 Office of Research Patient Care Services, Stanford Health Care, Menlo Park, CA USA; 3 Department of Primary Care and Population Health, School of Medicine, Stanford University, Stanford, USA; 4 Edwin S.H. Leong Centre for Healthy Children, Department of Pediatrics, The Hospital for Sick Children, University of Toronto, Toronto, ON Canada; 5 Institute of Health Policy, Management, and Evaluation, University of Toronto, Toronto, ON Canada; 6 Department of Pediatrics, Boston Children’s Hospital, Boston, MA USA; 7 Department of Clinical Epidemiology, Department of Clinical Medicine, Aarhus University and Aarhus University Hospital, Aarhus, Denmark

**Keywords:** Maternal Employment Status, Disability Pensions, Major Congenital Anomaly, Danish Workforce, Long-term Employment Consequences

## Abstract

**Importance:**

The burden of caring for children with complex medical problems such as major congenital anomalies falls principally on mothers, who in turn suffer a variety of potentially severe economic consequences. As well, health consequences of caregiving often further impact the social and economic prospects of mothers of children with major congenital anomalies (MCMCAs). Evaluating the long-term economic consequences of extensive in-home caregiving among MCMCAs can inform strategies to mitigate these effects.

**Objective:**

To assess whether MCMCAs face reduced employment and increased need for disability benefits over a 20-year period.

**Design:**

A population-based matched cohort study.

**Setting:**

Denmark.

**Participants:**

All women who gave birth to a singleton child with a major congenital anomaly in Denmark between January 1, 1997 and December 31, 2017 (*n* = 23,637) and a comparison cohort of mothers matched by maternal age, parity, and infant’s year of birth (*n* = 234,586).

**Exposures:**

Liveborn infant with a major congenital anomaly.

**Main outcomes and measures:**

The primary outcome was mothers’ employment status, stratified by their child’s age. Employment status was categorized as employed, outside the workforce (on temporary leave, holding a flexible job, or pursuing education), or unemployed; the number of weeks in each category was measured over time. The secondary outcome was time to receipt of a disability pension, which in Denmark implies permanent exit from the labor market. We used a negative binomial regression model to estimate the number of weeks in each employment category, stratified by the child’s age (*i.e.,* 0–1 year, > 1–6 years, 7–13 years, 14–18 years). A Cox proportional hazards regression model was used to compute hazard ratios as a measure of the relative risk of receiving a disability pension. Rate ratios and hazard ratios were adjusted for maternal demographics, pregnancy history, health, and infant’s year of birth.

**Results:**

During 1–6 years after delivery, MCMCAs were outside the workforce for a median of 50 weeks (IQR, 6–107 weeks), while members of the comparison cohort were outside the workforce for a median of 48 weeks (IQR, 4–98 weeks), corresponding to an adjusted rate ratio [ARR] of 1.05 (95% confidence interval [CI], 1.04–1.07). During the first year after delivery, MCMCAs were more likely to be employed than mothers in the comparison cohort (ARR, 1.08; 95% CI, 1.06–1.10). At all timepoints thereafter, MCMCAs had a lower rate of workforce participation. The rate of being outside the workforce was 5% higher than mothers in the comparison cohort during 1–6 years after delivery (ARR, 1.05; 95% CI, 1.04–1.07), 9% higher during 7–13 years after delivery (ARR, 1.09; 95% CI, 1.06–1.12), and 12% higher during 14–18 years after delivery (ARR, 1.12; 95% CI, 1.07–1.18). Overall, MCMCAs had a 20% increased risk of receiving a disability pension during follow-up than mothers in the matched comparison cohort [incidence rates 3.10 per 1000 person-years (95% CI, 2.89–3.32) vs. 2.34 per 1000 person-years (95% CI, 2.29–2.40), adjusted hazard ratio, 1.20; 95% CI, 1.11–1.29].

**Conclusion and relevance:**

MCMCAs were less likely to participate in the Danish workforce, less likely to be employed, and more likely to receive disability pensions than mothers of unaffected children. The rate of leaving the workforce intensified as their affected children grew older. The high demands of caregiving among MCMCAs may have long-term employment consequences even in nations with comprehensive and heavily tax-supported childcare systems, such as Denmark.

**Supplementary Information:**

The online version contains supplementary material available at 10.1186/s12889-024-18190-w.

## Background

Approximately 2%-6% of all infants born in the United States and Europe have major congenital anomalies (MCAs) [1–3]. Many of these children survive, but have chronic healthcare needs throughout their lifespan, hindering their ability to functional independently [4]. The burden of caring for children with chronic illness falls disproportionately on mothers [5], who in turn suffer a variety of health and economic consequences [6, 7]. Compared to other mothers, mothers of children with major congenital anomalies (MCMCAs) are sicker, have a shorter lifespan [7], and use healthcare services more often [8].

The health consequences of caregiving often also impact MCMCAs' economic prospects [9]. Studies have reported that parents of chronically ill children are more likely to reduce work hours [10, 11], take sick days [12], and experience unemployment [13, 14]. However, these studies have important limitations. They included groups of children with varying levels of need, from mild to severe [11, 12, 15, 16], and relied on old data ranged from 2001 to 2006 [11, 12]. Some lacked comparison groups [11] and were unable to assess long-term outcomes [14].

The “motherhood penalty” has been well-described in many high income countries, where mothers with children tend to have lower employment rates, work fewer hours, and earn less than other women [17–21]. This penalty has been found to range from 4 to 8% [18, 19]. However, the extent to which this “penalty” is exacerbated for mothers of children with substantial medical needs remains unknown [22]. We therefore examined the relation between MCMCA status, employment, and receipt of disability pensions in a single country over nearly two decades.

## Methods

### Study design, setting, and data

We conducted a population-based cohort study in Denmark, which provides universal tax-supported healthcare [23, 24] and various comprehensive forms of support for families with children are available for all citizens. These include 52 weeks of paid parental leave [25], guaranteed access to day care [26], and maternal financial support [27], among other social services [28, 29].

The Danish Civil Registration System assigns a unique personal registry identifier to all Danish residents [30], which we used to link to four national registries to define and obtain information about our cohorts of mothers. These registries included the Danish Medical Birth Registry, the Danish National Patient Registry, the Danish Register for Evaluation of Marginalisation (DREAM) database, and Danish National Prescription Registry data [31]. A detailed description of these registries is provided in the Supplement (eStudy Methods).

The study was reported to the Danish Data Protection Agency (2016–051-000001 / 605); informed consent and ethics approval were not required according to Danish law. The study was reported according to the Strengthening the Reporting of Observational Studies in Epidemiology (STROBE) guidelines (eTable 1).

### Study cohorts

Our analytic cohort included 23,637 women who had given birth to a live singleton with an MCA (the “exposed cohort”) between January 1, 1997 and December 31, 2017. Infants with MCA were identified using ICD-10 codes in accordance with the European Surveillance of Congenital Anomalies Classification System (eTable 2). For mothers who gave birth to more than one child with an MCA, we considered the first affected pregnancy as the index pregnancy. A matched population-based cohort of 234,586 women was selected as the “comparison cohort”. We used 10:1 random sampling with replacement [32] and matched on maternal age at delivery, parity (1, 2, and $\ge$ 3 children), and infant’s year of birth. We followed these women until one of the following occurred: December 31, 2018; emigration; the child’s 18th birthday; loss to follow up; or death.

**Fig. 1:**
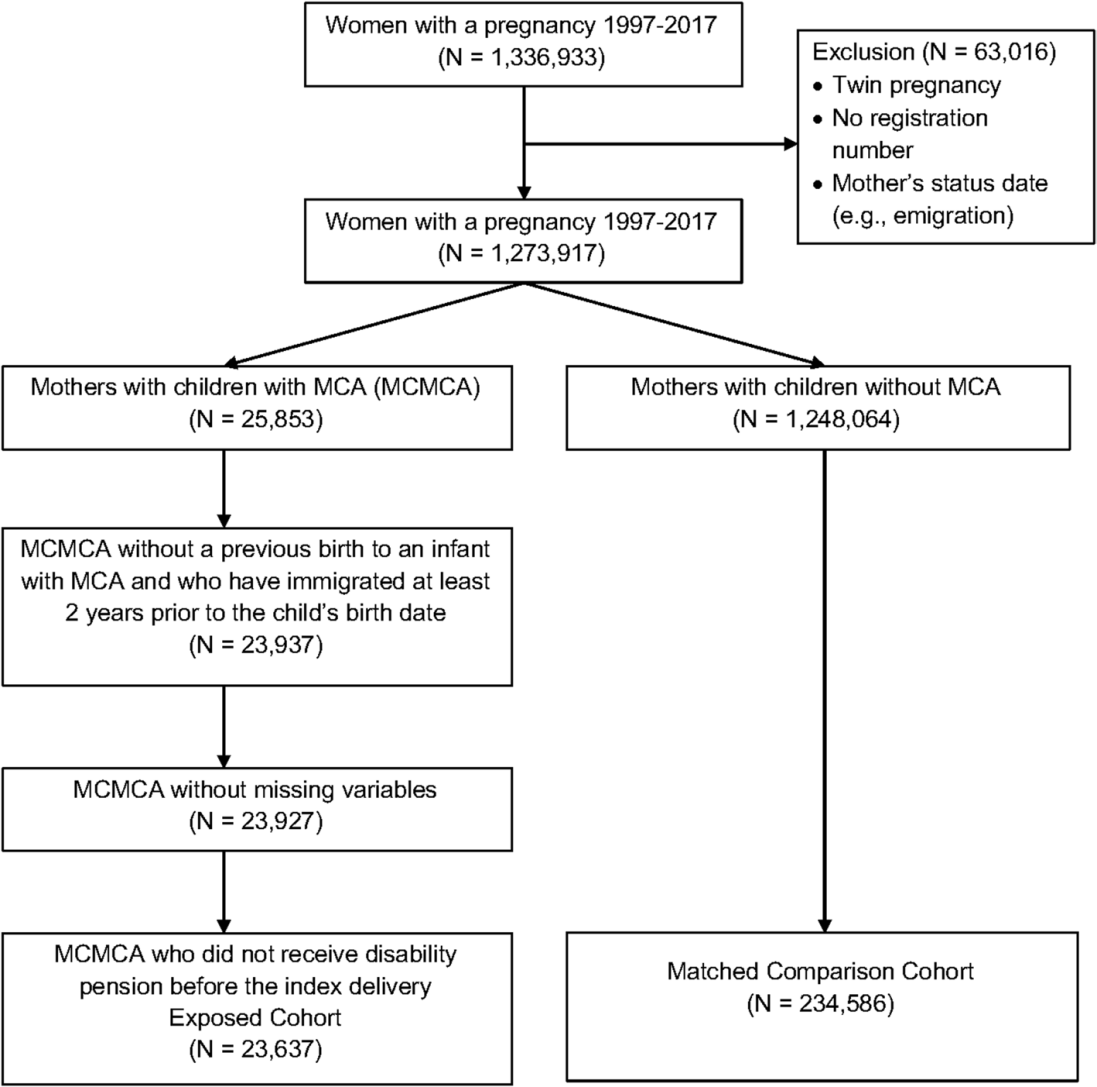
Flow diagram of cohort selection

Mothers were excluded who (1) lacked a minimum of two years of data prior to giving birth, (2) had missing values for matching or key study variables, and (3) or received a disability pension before the index delivery. Applying these criteria resulted in the exclusion of 2218 (8.6%) of MCMCA (Fig. 1).

### Study outcomes

The primary outcome was employment status, stratified into 4 groups based on child age categories that align with the 52 weeks of paid parental leave currently provided to mothers in Denmark [25] and the age cutoffs of the Danish educational system (0–1 year, > 1–6 years [pre-school], 7–13 years, [primary education], 14–18 years [secondary education or higher]).

We created a three-category variable to measure employment status: employed (those currently working), unemployed (those receiving unemployment benefits or job training, but remaining in the job market), or outside the workforce (those on temporary leave; holding a flexible job; or pursuing education) (eTable 3). We measured employment status in a given period as the number of weeks in each employment category.

The secondary outcome was time to receipt of a disability pension. Persons who are disabled for longer periods (and thus are no longer able to maintain a regular or flex job) receive an early retirement disability pension and permanently exit the labor market in Denmark.

### Covariates

We selected the following characteristics, associated a priori with employment status, as covariates: maternal demographics (age and marital status at time of the index delivery, income quartile and level of education ascertained the year prior to the index birth, and immigration status), pregnancy history (parity and pregnancy-related health conditions), pre-existing comorbid illness status (Charlson Comorbidity Index (CCI) score, pre-birth mental illness), and infant’s year of birth. A list of ICD-8 and ICD-10 diagnostic codes used to determine the CCI score and the World Health Organization Anatomical Therapeutic Chemical Classification codes used to identify prescriptions for pre-birth mental illness is summarized in eTable 2. Since information on body mass index (BMI) became available after the beginning of the study period (2004), BMI data were used only in a sensitivity analysis.

### Statistical analysis

We examined the annual number of weeks in each employment category from 2 years prior to delivery up to 18 years after delivery. To evaluate the unadjusted and adjusted association between MCMCA status and employment, we used a negative binomial regression model with a robust variance estimator to calculate the rate ratio (RR) of the number of weeks in each employment status category, stratified by the child’s age. To assess the association between MCMCA status and receipt of a disability pension, we computed crude incidence rates. We then used Cox proportional hazards regression analysis to compute hazard ratio (HR) with 95% confidence interval (CI) as a measure of relative risk of receiving a disability pension. The proportional hazards assumption was assessed and confirmed using log–log plots. The follow-up started at the date of birth.

We conducted stratified analyses by the listed covariates, including maternal health status, education level, and year of delivery, comparing the exposed cohort to the comparison cohort. To examine a potential dose–response association between exposure and outcome, we stratified the main model by the number of affected organ systems in the child (*i.e*., single organ vs. multiple organ anomalies).

We conducted several sensitivity analyses. First, we assessed the robustness of our main results by examining different ways of assessing employment status using labor market participation. The “employed” category was defined as participation in the labor market during at least 50%, 75%, or 90% of a given period. We compared the percentage of mothers whose labor market participation was greater than these thresholds between the exposed and the comparison cohorts, using modified Poisson regression models with robust variance estimators, accounting for repeated measures for the same individual. To account for unobserved factors related to labor market attachment prior to delivery, we conducted two sensitivity analyses. We repeated the main analyses solely with mothers who were employed in the year prior to the delivery year. In this sensitivity analysis, we counted the total weeks spent in each employment category, identifying the category where the most time was spent as the employment status for the period before delivery. Next, we excluded mothers whose participation in the labor market was below 75% one year before beginning maternity leave or one year before the delivery date for those not receiving maternity leave benefits. During the 2004–2017 period, we further adjusted the models for BMI. To address the potential impact of the disability pension policy reform put in place on January 1, 2013, we conducted four additional sensitivity analyses. The reform prevented persons under the age of 40 years from receiving a disability pension and instead provided them with vocational training and flexible jobs [33]. In the first policy reform-related sensitivity analysis, we stratified the main analysis by the year of policy implementation (*i.e*., before and after 2013). We then examined the risk of receiving a disability pension by treating vocational training and flexible jobs as equivalent to a disability pension starting in 2013. We also estimated the risk of receiving a disability pension by defining vocational training and flexible jobs as equivalent to a disability pension for the entire study period. Finally, we censored follow-up time on December 31, 2012, for those who gave birth before the change in the disability pension policy and computed HR.

All data analyses were performed using SAS 9.4 (SAS Institute, Cary, NC).

## Results

### Characteristics of study cohorts

Most mothers were married and employed, and median age at the index delivery was 30 years (interquartile range [IQR], 27–34 years) (Table 1). Compared to the comparison cohort, exposed mothers were less educated (bachelor degree or higher, 29.4% vs. 31.8%), more likely to be unmarried (46.5% vs. 43.7%), had slightly more comorbidities (CCI score greater than 2, 1.6% vs. 1.2%), and had more pre-birth histories of mental health diagnoses (5.8% vs. 4.9%). Among children with MCAs, mortality during the first year was 0.5% for single-organ MCA and 2.2% for multiple-organ MCA.

**Table 1:** Characteristics of mothers included in the exposed and comparison cohorts and their infants

Characteristics^a^	Mothers with a child with major congenital anomalies No. (%)	Mothers with a child without major congenital anomalies No. (%)
***Mothers***	23,637	234,586
Age at index delivery		
≤ 25 years	3356 (14.2)	33,543 (14.3)
26–35 years	15,615 (66.1)	155,329 (66.2)
36 + years	4666 (19.7)	45,714 (19.5)
Parity		
1	11,179 (47.3)	111,129 (47.4)
2	8085 (34.2)	80,398 (34.3)
≥ 3	4373 (18.5)	43,059 (18.4)
Marital status^b^		
Married/Registered partnership	12,614 (53.4)	126,563 (54.0)
Single/Divorced/Widowed/Living alone	10,993 (46.5)	102,496 (43.7)
Unknown	30 (0.1)	5527 (2.4)
Income quartile		
≤ 25%	6046 (25.6)	58,369 (24.9)
26–50%	6146 (26.0)	58,387 (24.9)
51–75%	5880 (24.9)	58,721 (25.0)
76–100%	5533 (23.4)	58,855 (25.1)
Missing	32 (0.1)	254 (0.1)
Highest education attained		
Primary or secondary	8088 (34.2)	76,115 (32.4)
Vocational or post-secondary	7675 (32.5)	74,937 (31.9)
Bachelor’s degree or higher	6952 (29.4)	74,611 (31.8)
Missing	922 (3.9)	8923 (3.8)
Pregnancy history		
Spontaneous abortions	3967 (16.8)	35,385 (15.1)
Stillbirths	10 (0.0)	22 (0.0)
Index pregnancy complications		
Placental	1649 (7.0)	10,957 (4.7)
Non-placental	193 (0.8)	930 (0.4)
Multiple pregnancies with MCAs during follow-up		
Single MCA pregnancy	23,097 (97.7)	-
Multiple MCA pregnancies	540 (2.3)	-
Medical history using ICD-8/10 diagnostic codes		
Diabetes	314 (1.3)	1303 (0.6)
Alcohol-related diseases	400 (1.7)	3145 (1.3)
Any pre-birth mental health diagnosis	1368 (5.8)	11,541 (4.9)
Any mental health diagnoses during follow up	1662 (7.0)	12,898 (5.5)
Medical history using prescription data^c^ as well as ICD-8/10 diagnostic codes		
Diabetes	824 (3.5)	4959 (2.1)
Any pre-birth mental health diagnosis	4119 (17.4)	35,550 (15.2)
Any mental health diagnoses during follow up	6679 (28.3)	58,239 (24.8)
Charlson Comorbidity Index Score		
0	21,975 (93.0)	220,254 (93.9)
1	1290 (5.5)	11,595 (4.9)
≥ 2	372 (1.6)	2737 (1.2)
Immigrated	2866 (12.1)	27,329 (11.6)
Maternal BMI (2004–2017)		
< 25	9528 (60.2)	101,416 (64.5)
25–29	3345 (21.1)	31,798 (20.2)
≥ 30	2334 (14.7)	18,758 (11.9)
Duration of follow-up, median (IQR), years	11.3 (5.9–16.5)	11.3 (5.9–16.5)
Employment status^d^		
Employed	14,291 (60.5)	147,094 (62.7)
Outside workforce	7539 (31.9)	70,178 (29.9)
Unemployed	1605 (6.8)	15,316 (6.5)
***Infant***		
Sex		
Male	13,971 (59.1)	119,826 (51.1)
Female	8563 (36.2)	113,815 (48.5)
Birthweight, g		
≤ 2500	3589 (15.2)	8494 (3.6)
> 2500–4000	16,736 (70.8)	184,736 (78.7)
> 4000	3192 (13.5)	40,693 (17.3)
Gestational age, weeks		
15–36	3864 (16.3)	11,209 (4.8)
37–41	18,563 (78.5)	209,198 (89.2)
42–50	1074 (4.5)	12,992 (5.5)
Apgar score < 7 at 5 min	1269 (5.4)	3376 (1.4)
Year of infant's birth		
1997–2003	7798 (33.0)	77,460 (33.0)
2004–2010	7868 (33.3)	78,090 (33.3)
2011–2017	7971 (33.7)	79,036 (33.7)
Severity of MCA		
Single-organ MCA	21,101 (89.3)	-
Multiple-organ MCA	2536 (10.7)	-
Number of hospitalizations within the first year of life		
0 hospitalization	6307 (26.7)	182,345 (77.7)
1–3 hospitalizations	12,552 (53.1)	49,862 (21.3)
4 + hospitalizations	4778 (20.2)	2379 (1.0)
Infant death by 1 year	155 (0.7)	60 (0.0)
Single-organ MCA	99 (0.5)	-
Multiple-organ MCA	56 (2.2)	-
Mortality with single-organ MCA		
at 0–1 year	99 (0.5)	-
at > 1–6 years	121 (0.6)	-
at 7–13 years	25 (0.1)	-
at 14–18 years	16 (0.1)	-
Mortality with multiple-organ MCA		
at 0–1 year	56 (2.2)	-
at > 1–6 years	68 (2.7)	-
at 7–13 years	16 (0.6)	-
at 14–18 years	5 (0.2)	-
Mortality with no hospitalizations within the first year of life		
at 0–1 year	30 (0.5)	22 (0.0)
at > 1–6 years	11 (0.2)	79 (0.0)
at 7–13 years	< 5	Cannot be presented^e^
at 14–18 years	< 5	Cannot be presented^e^
Mortality with 1–3 hospitalizations within the first year of life		
at 0–1 year	66 (0.5)	29 (0.1)
at > 1–6 years	59 (0.5)	31 (0.1)
at 7–13 years	14 (0.1)	12 (0.0)
at 14–18 years	9 (0.1)	13 (0.0)
Mortality with > 3 hospitalizations within the first year of life		
At 0–1 year	59 (1.2)	9 (0.4)
at > 1–6 years	119 (2.5)	21 (0.9)
at 7–13 years	Cannot be presented^e^	< 5
at 14–18 years	Cannot be presented^e^	< 5

^a^All maternal characteristics are at baseline except the following: income quartile and level of education in the year prior to the index delivery, multiple pregnancies with MCAs during follow-up, any mental health diagnoses during follow-up, and depression diagnoses during follow-up

^b^Marital status denotes legal marital status. Those who are unmarried but living with a partner without registration would be categorized as single

^c^Prescription data were obtained from the Danish National Prescription Registry using World Health Organization Anatomical Therapeutic Chemical Classification codes, which are available in eTable 2 of the Supplement. In the main analysis, mental illnesses identified from both ICD8/10 codes and prescription data were adjusted, while only diabetes identified using ICD 8/10 codes was adjusted as part of the Charlson Comorbidity Index score

^d^The employment status presented in Table 1 is based on the social benefits received during the 52 weeks prior to delivery

^e^The frequency for this cell was 5 or above. However, values cannot be presented due to the possibility of back-calculation of sensitive data

### Association between MCMCA status and employment

During 1–6 years after delivery, MCMCAs had a median of 149 weeks of employment (IQR, 51–215 weeks), while comparison cohort members were employed for a median of 155 weeks (IQR, 58–217 weeks), corresponding to an unadjusted rate ratio of 0.97 (95% CI, 0.96–0.98). In contrast, during the same period MCMCAs were outside the workforce for a median of 50 weeks (IQR, 6–107 weeks) *vs.* a median of 48 weeks (IQR, 4–98) weeks for the comparison cohort (unadjusted RR, 1.07; 95% CI, 1.06–1.09).

**Fig. 2:**
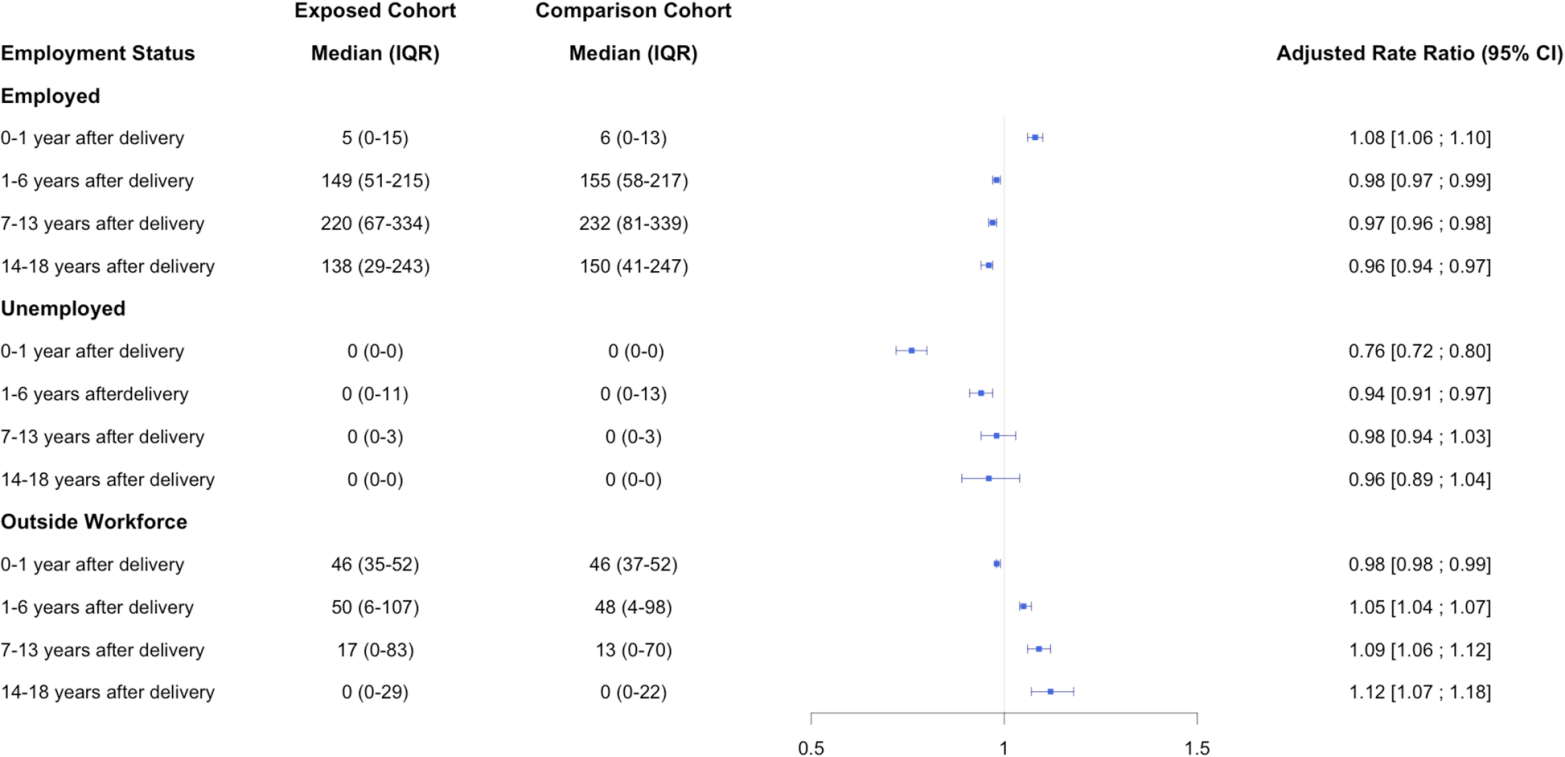
Covariate-adjusted^a^ association between mothers of children with major congenital anomalies and employment status. ^a^Adjusted covariates included maternal demographics (age at delivery, marital status, income quartile, level of education ascertained as of the year prior to the index birth, and immigration status), pregnancy history (parity and pregnancy-related health conditions), and health (Charlson Comorbidity Index score, pre-birth mental illness)

The covariate-adjusted analyses showed that MCMCAs were more likely to be employed than mothers in the comparison cohort (adjusted RR [ARR], 1.08; 95% CI, 1.06–1.10) during the first year after delivery when all women are entitled to maternity leave. However, MCMCAs were more likely to be outside the workforce thereafter: rates of being outside the workforce were 5% higher during 1–6 years after delivery (ARR, 1.05; 95% CI, 1.04–1.07), 9% higher during 7–13 years after delivery (ARR, 1.09; 95% CI, 1.06–1.12), and 12% higher during 14–18 years after delivery (ARR, 1.12; 95% CI, 1.07–1.18) (Fig. 2).

### Association between MCMCAs and receiving a disability pension

**Fig. 3:**
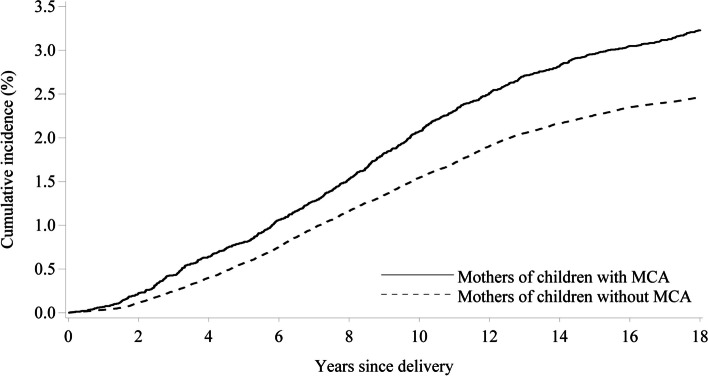
Cumulative incidences of disability pension receipt among mothers of children with and without major congenital anomalies. Note: The follow-up for the Cox regression started at the date of birth

Crude incidence rates of receiving a disability pension were 3.10 per 1000 person-years for mothers in the exposed cohort (95% CI, 2.89–3.32) and 2.34 per 1000 person-years for members of the comparison cohort (95% CI, 2.29–2.40), corresponding to an unadjusted HR of 1.33 (95% CI, 1.23–1.43). Overall, MCMCAs had a 20% increased risk of receiving a disability pension after delivery (adjusted HR 1.20; 95% CI, 1.11–1.29) (Fig. 3).

### Stratified analyses

In the stratified analyses, compared to the comparison cohort, MCMCAs without psychiatric history or pregnancy-related complications were more likely to be outside the workforce, whereas MCMCAs with these conditions had a similar rate of being outside the workforce than the comparison cohort (eTables 4, 5). Overall, the rate of being outside the workforce was similar among MCMCAs with and without comorbidity (eTable 6). Similarly, MCMCAs without psychiatric history had a higher risk of receiving a disability pension, while MCMCAs with such a history had a risk level similar to the comparison cohort (eTable 7). When stratified by comorbidity, MCMCAs at all CCI score levels were at a greater risk of receiving a disability pension compared to the comparison cohort. Those with the most severe comorbidity scores, that is a CCI score of 2 or more, had the highest relative risk (eTable 8).

While the rate of being outside the workforce was higher in the MCMCA cohort compared to the comparison cohort, this difference did not vary based on education level or the number of affected organ systems in the child (eTables 9, 10).

Analyses stratified by year of delivery between 1997 and 2005 yielded results consistent with those of the main analyses. Of note, in nearly all years between 2006 and 2017 the likelihood of being employed during the first year after delivery did not differ between the exposed and comparison cohorts (eTable 11).

### Sensitivity analyses

The results of the sensitivity analyses were consistent with those for the main analyses across the study outcomes, indicating decreased employment among MCMCAs using various cut points for labor market participation (eTables 12–14). When we additionally adjusted for BMI in the years 2004–2017, the results largely reflected those obtained without controlling for BMI (eTable 15).

When we stratified the analysis by the year of disability pension policy reform, the results were consistent for the years 1997–2012, indicating increased employment among MCMCAs during the first year after delivery but more likely to being outside the workforce thereafter. However, for years 2013–2017, the rates of employment were similar in both the MCMCA and comparison cohorts during the first year following delivery (eTable 16). Results from the sensitivity analyses, which incorporated vocational training and flexible jobs into the definition of receiving a disability pension, were consistent with our main findings (eTable 17).

## Discussion

Our study showed that MCMCAs in Denmark face small but consistently intensifying social and economic sequelae from caregiving over time. MCMCAs were less likely to be employed and more likely to receive a disability pension than were mothers in an unexposed comparison cohort. We also found that marginalization from the labor market intensifies as children with MCAs age. Our findings are consistent with limited prior evidence concerning the economic burdens borne by families with chronically ill children [22]. They illustrate that the long-term demands of caring for sick children, which can force these mothers to leave the workforce—even in a system as comprehensive as Denmark’s, where a robust and heavily tax-subsidized childcare system is in place [34].

Factors related to the employment status of mothers of chronically ill children are complex. We found that MCMCAs were less likely to participate in the workforce compared to their matched peers. This pattern persisted throughout an extensive follow-up period over 20 years, except for the first year after delivery, as discussed further below. The care required for children with MCAs involves not only direct care-related responsibilities, such as providing care to a child with complex needs at home and managing surgical and rehabilitative care, but also learning about these conditions and managing them. Mothers are also tasked with navigating the healthcare system, from obtaining a diagnosis and accessing specialist care, to seeking social support and dealing with the associated stigma [35]. These complex care needs contributed to these mothers staying outside of the workforce long-term.

We also found that MCMCAs had an increased likelihood of being employed during the first year after delivery, except for the period of 2013–2017, which showed similar rates of employment. This may appear contradictory, given that all mothers of newborns in Denmark are entitled to maternity leave, and given the toll of caregiving for children with MCAs in early life. A potential explanation for this finding would be that that individuals with higher financial caregiving burdens may initially increase their levels of employed work, before decreasing employment due to increasingly time-intensive burdens of caregiving for school-age children with ongoing health challenges [11]. Our findings are consistent with prior evidence showing bimodal employment patterns for mothers of sick children, indicated in studies from the United States in the 2000s. [11, 14, 35, 36]. While the results observed in the US setting may not be directly comparable to the Danish context due to differences in social welfare systems, increased employment during the first year was a consistently observed pattern in our comprehensive sensitivity analysis. This was the case even when we applied different labor market participation thresholds and excluded mothers who had not participated in the labor market before delivery, among other considerations.

Another potential reason for the increased employment status of MCMCAs during the first year might be that these mothers, despite caring for children with MCAs, may not be providing full-time childcare. If they continue to earn wages, such as from their employers, even during the 52 weeks of maternity/parental leave that Danes are entitled to, they could be classified as either employed or on maternity leave. Consequently, our employment estimates for the first year after delivery might be overestimated. Such an artifact of data collection could help explain why employment status in the first year contrasts that of all subsequent years thereafter, and would support our overall conclusions about the impact of being an MCMCA on labor market participation.

Our findings build upon previous research, suggesting that having a child with MCAs is not only associated with deteriorating maternal health, but it also contributes to the mother's eventual withdrawal from the workforce due to severe health conditions, namely disability. Several explanations may exist for this observation. First, having a child with MCAs might exacerbate pre-existing physical conditions in mothers, rendering them unable to stay in the workforce. Second, acute maternal health deterioration associated with the delivery of MCAs (e.g., maternal surgery required due to the delivery of a child with MCA) might seriously impair these mothers' health conditions. Third, the onset of mental health issues among MCMCAs, reported to be particularly prevalent during the first year postpartum [37], could explain the increased risk of receiving disability pensions during the early years post-delivery among MCMCAs, given that mental health issues are one of the primary reasons for granting disability pensions in Denmark [33]. Fourth, the long-term mental health strain associated with caring for a child with MCA could increase the risk of receiving a disability pension, considering the increase in mental health utilization over time as reported in a previous study [8].

Our findings have important policy implications. Postpartum social supports in the United States, such as the Special Supplementation Nutrition Program for Women, Infants, and Children [38], mainly focus on the first few years of a child’s life, neglecting the intensifying needs of mothers of sick children over time. In contrast, our findings collectively suggest that mothers of chronically ill children may need ongoing multi-dimensional support, varying by stage of child development, to maintain their participation in or re-entry into the workforce, in terms of finances, healthcare, and respite alike.

The repercussions of caregiving are not limited to mothers. The burdens impact the whole family, including fathers and siblings of chronically ill children [39, 40], as well as the greater society. The societal burdens of caregiving can be measured in loss of productivity for persons staying out of the workforce, decreased productivity among those who stay at work, increased hiring costs for businesses, and lost tax revenues [41]. Future studies of societal costs attributable to mothers with chronic and intense caregiving burdens are necessary to enumerate the cost-benefits of potential interventions to support these mothers and to design evidence-informed policies.

### Limitations

Our study has several limitations. First, our classification of employment status has not been validated, and our results might be biased by misclassification of employment status. However, selected codes for employment [42] and sick leave records [43] in the DREAM database were previously validated. Furthermore, to address this concern, we used a variable available beginning in 2008 that indicates whether members of the study cohorts had a wage income. This allowed us to cross-check employment status among those having a wage income. We also found consistent results in our sensitivity analyses, when employment status was defined using labor market participation scores. The effect sizes also were greater. A second concern is that the observed associations between MCMCAs and employment status or receiving the disability pension might be overestimated due to confounders such as the mother’s own underlying health conditions prior to the index delivery, education level, and behavioral aspects such as smoking, which could potentially decrease workforce attachment. To address this issue, adjustments were made for as many measurable maternal factors as possible that were included in the dataset. We also carefully examined these factors between the two cohorts before delivery and observed no substantial pre-existing differences in these conditions between two cohorts prior to the birth. Additionally, we conducted stratified analyses to compare the employment status between the MCMCA cohort and the comparison cohort within those with similar confounding characteristics. Finally, we performed a sensitivity analysis excluding mothers who did not participate in the labor force prior to delivery, in order to exclude those who were likely to have certain conditions related to low attachment to the labor force. These analyses confirmed the robustness of our main results. A third limitation was our inability to access employer-sponsored short-term sick leave data, which could cause underestimation of the number of mothers outside the workforce. However, such underestimation is likely minimal because persons outside the workforce for longer periods would transition to government-sponsored disability benefits [44]. A fourth limitation was omission of the role of spouse/domestic partner in influencing maternal employment status. Finally, our results apply to the Danish population. Because our study took place [23, 45] in a country providing one of the most established welfare and social support systems, our findings may underestimate the negative impact of high caregiving burdens on maternal employment in countries with fewer supports, such as the United States.

## Conclusion

We found an increased rate of workforce non-participation and receipt of disability pensions among mothers of children born with major anomalies. These risks accumulated as the child aged. Understanding the downstream challenges faced by caregiving mothers can inform development of evidence-based policy initiatives to mitigate these challenges and to sustain workforce participation, thereby improving individual wellbeing and increasing overall social productivity.

### Supplementary Information

**Supplementary Material 1.**

## Data Availability

Danish law does not allow researchers to share raw data from the registries with third parties. Data can be accessed by researchers through application to the Danish Data Protection Agency (dt@datatilsynet.dk) and the Danish Health Data Authority (kontakt@sundhedsdata.dk).

## Abbreviations

BMI: Body mass index
CCI: Charlson Comorbidity Index
CI: Confidence interval
DREAM: The Danish National Patient Registry, the Danish Register for Evaluation of Marginalisation
HR: Hazard ratio
ICD: International Statistical Classification of Diseases and Related Health Problems
IQR: Interquartile range
MCA: Major congenital anomaly
MCMCA: Mothers of children with major congenital anomaly
RR: Rate ratio
STROBE: The Strengthening the Reporting of Observational Studies in Epidemiology
