# Supplementary material for: Association between maternal employment status and presence of children with major congenital anomalies in Denmark
Source: BMC Public Health. 2024 Mar 6;24:715. doi: 10.1186/s12889-024-18190-w (PMC10916273; doi:10.1186/s12889-024-18190-w)
Supplement: Supplementary file 1 — Supplementary Material 1. [file 12889_2024_18190_MOESM1_ESM.docx]

**Supplements**

**eStudy Methods.** Description of the Danish Registry Data

**eTable 1.** STROBE Statement Checklist

**eTable 2.** Diagnostic Codes Used in the Study with Relevant References

**eTable 3.** List of Dream Codes Used to Measure Employment Status

**eTable 4.** Stratified Analysis: Rate of Being Outside Workforce in the Mothers of Children with Major Congenital Anomalies Cohort by Pre-Pregnancy Psychiatric Medical History compared to the Matched Comparison Cohort

**eTable 5.** Stratified Analysis: Rate of Being Outside Workforce in the Mothers of Children with Major Congenital Anomalies Cohort by Pregnancy-related Complications Index compared to the Matched Comparison Cohort

**eTable 6.** Stratified Analysis: Rate of Being Outside Workforce in the Mothers of Children with Major Congenital Anomalies Cohort by Charlson Comorbidity Index (CCI) compared to the Matched Comparison Cohort

**eTable 7.** Stratified Analysis: Rate of Receiving Disability Pension in the Mothers of Children with Major Congenital Anomalies Cohort by Pre-Pregnancy Psychiatric Medical History compared to the Matched Comparison Cohort

**eTable 8.** Stratified Analysis: Rate of Receiving Disability Pension in the Mothers of Children with Major Congenital Anomalies Cohort by Charlson Comorbidity Index (CCI) compared to the Matched Comparison Cohort

**eTable 9.** Stratified Analysis: Rate of Being Outside Workforce in the Mothers of Children with Major Congenital Anomalies Cohort by Education compared to the Matched Comparison Cohort

**eTable 10.** Stratified Analysis: Rate of Being Outside Workforce in the Mothers of Children with Major Congenital Anomalies Cohort by Affected Organ System compared to the Matched Comparison Cohort

**eTable 11.** Stratified Analysis: Rate of Being Employed in the Mothers of Children with Major Congenital Anomalies Cohort by the year of delivery compared to the Matched Comparison Cohort

**eTable 12.** Sensitivity Analysis: Examining employment status using various thresholds of labor market participation, “employed” referred to those who participated in the labor market during at least 50%, 75%, or 90% of the given period.

**eTable 13.** Sensitivity Analysis: Rate of Being Outside Workforce in the Mothers of Children with Major Congenital Anomalies Cohort compared to the Matched Comparison Cohort, Excluding Non-Active Labor Market Participation Prior to Delivery.

**eTable 14.** Sensitivity Analysis: Employment Status of Mothers with At Least 75% Labor Market Participation, Excluding Non-Active Labor Market Participation Prior to Delivery.

**eTable 15.** Sensitivity Analysis: The Mothers of Children with Major Congenital Anomalies Cohort vs. the Matched Comparison Cohort for Body Mass Index in the 2004-2017 period

**eTable 16.** Sensitivity Analysis: Association between MCMCAs and Employment Status, Stratified by Year 2013

**eTable 17.**  Sensitivity Analysis: Association between being MCMCA and Receiving Disability Pension

**eFigure 1.** Unadjusted Annual Mean weeks in Employment

**eFigure 2.** Unadjusted Annual Mean weeks in Outside Workforce

**eFigure 3.** Unadjusted Annual Mean weeks in Unemployment

**eStudy Methods. Description of the Danish Registry Data**

The Danish Civil Registration System allowed us to obtain complete demographic data and perform individual-level data linkage across the following data sources:^1^ the Danish Medical Birth Registry for identification of all women who gave birth to a live singleton;^2^ the Danish National Patient Registry (DNPR) covering all Danish hospitals to identify cohorts of children with major congenital anomalies and ascertain maternal medical history;^3^ and the Danish Register for Evaluation of Marginalisation (DREAM) database, which provides information on employment and social benefit use (e.g., on a weekly basis and workforce-related benefit use, such as sick leave and receipt of disability pensions.^4–6^ We also used Danish National Prescription Registry data^7^ to identify prescriptions for psychiatric disorder and diabetes. This Registry allowed us to improve data capture for a sensitivity analysis, since coverage of these conditions in the DNPR historically has been lower than reported in the previous literature.^8–11^

**References**

1. Schmidt M, Schmidt SAJ, Adelborg K, et al. The Danish health care system and epidemiological research: from health care contacts to database records. *CLEP*. 07/2019;11:563-591.

2. Bliddal M, Broe A, Pottegård A, Olsen J, Langhoff-Roos J. The Danish Medical Birth Register. *Eur J Epidemiol*. 2018;33(1):27-36.

3. Schmidt M, Schmidt SAJ, Sandegaard JL, Ehrenstein V, Pedersen L, Sørensen HT. The Danish National Patient Registry: a review of content, data quality, and research potential. *Clin Epidemiol*. 2015;7:449-490.

4. Stapelfeldt CM, Jensen C, Andersen NT, Fleten N, Nielsen CV. Validation of sick leave measures: self-reported sick leave and sickness benefit data from a Danish national register compared to multiple workplace-registered sick leave spells in a Danish municipality. *BMC Public Health*. 2012;12(1). doi:10.1186/1471-2458-12-661

5. Skajaa N, Adelborg K, Horváth-Puhó E, et al. Labour market participation and retirement after stroke in Denmark: registry based cohort study. *BMJ*. 2023;380:e072308.

6. Hjollund NH, Larsen FB, Andersen JH. Register-based follow-up of social benefits and other transfer payments: Accuracy and degree of completeness in a Danish interdepartmental administrative database compared with a population-based survey. *Scand J Public Health*. 10/2007;35(5):497-502.

7. Pottegård A, Schmidt SAJ, Wallach-Kildemoes H, Sørensen HT, Hallas J, Schmidt M. Data Resource Profile: The Danish National Prescription Registry. *Int J Epidemiol*. 2017;46(3):798-798f.

8. Carstensen B, Rønn PF, Jørgensen ME. Components of diabetes prevalence in Denmark 1996–2016 and future trends until 2030. *BMJ Open Diabetes Research and Care*. 2020;8(1):e001064.

9. Højlund M, Andersen JH, Andersen K, Correll CU, Hallas J. Use of antipsychotics in Denmark 1997–2018: a nation-wide drug utilisation study with focus on off-label use and associated diagnoses. *Epidemiol Psychiatr Sci*. 2021;30. doi:10.1017/S2045796021000159

10. Kosjerina V, Carstensen B, Jørgensen ME, et al. Discontinuation of diabetes medication in the 10 years before death in Denmark: a register-based study. *The Lancet Healthy Longevity*. 2021;2(9):e561-e570. doi:10.1016/s2666-7568(21)00170-7

11. Sundbøll J, Adelborg K, Mansfield KE, Tomlinson LA, Schmidt M. Seventeen-Year Nationwide Trends in Antihypertensive Drug Use in Denmark. *Am J Cardiol*. 2017;120(12):2193-2200.

**eTable 1.** STROBE Statement Checklist

The study followed the Strengthening the Reporting of Observational Studies in Epidemiology (STROBE) reporting guidelines. Details are provided below.

|  | **Item No** | **Recommendation** |  | **Page** |
| --- | --- | --- | --- | --- |
| **Title and abstract** | 1 | (*a*) Indicate the study’s design with a commonly used term in the title or the abstract |  | 3 |
|  |  | (*b*) Provide in the abstract an informative and balanced summary of what was done and what was found |  | 3 |
| **Introduction** | | |  |  |
| Background/rationale | 2 | Explain the scientific background and rationale for the investigation being reported |  | 5 |
| Objectives | 3 | State specific objectives, including any prespecified hypotheses |  | 5 |
| **Methods** | | |  |  |
| Study design | 4 | Present key elements of study design early in the paper |  | 6 |
| Setting | 5 | Describe the setting, locations, and relevant dates, including periods of recruitment, exposure, follow-up, and data collection |  | 6-7 |
| Participants | 6 | (*a*) Give the eligibility criteria, and the sources and methods of selection of participants. Describe methods of follow-up |  | 6-7 |
|  |  | (*b*) For matched studies, give matching criteria and number of exposed and unexposed |  | 7 |
| Variables | 7 | Clearly define all outcomes, exposures, predictors, potential confounders, and effect modifiers. Give diagnostic criteria, if applicable |  | 7-8 |
| Data sources/ measurement | 8* | For each variable of interest, give sources of data and details of methods of assessment (measurement). Describe comparability of assessment methods if there is more than one group |  | 6,  eStudy Methods |
| Bias | 9 | Describe any efforts to address potential sources of bias |  | 9, 15, 17 |
| Study size | 10 | Explain how the study size was arrived at |  | 6-7 |
| Quantitative variables | 11 | Explain how quantitative variables were handled in the analyses. If applicable, describe which groupings were chosen and why |  | 7-8 |
| Statistical methods | 12 | (*a*) Describe all statistical methods, including those used to control for confounding |  | 8-10 |
|  |  | (*b*) Describe any methods used to examine subgroups and interactions |  | 9 |
|  |  | (*c*) Explain how missing data were addressed |  | 7 |
|  |  | (*d*) If applicable, explain how loss to follow-up was addressed |  | 7 |
|  |  | (*e*) Describe any sensitivity analyses |  | 9-10 |
| **Results** | | |  |  |
| Participants | 13* | (a) Report numbers of individuals at each stage of study—eg numbers potentially eligible, examined for eligibility, confirmed eligible, included in the study, completing follow-up, and analysed |  | Figure 1 |
|  |  | (b) Give reasons for non-participation at each stage |  | Figure 1 |
|  |  | (c) Consider use of a flow diagram |  | Figure 1 |
| Descriptive data | 14* | (a) Give characteristics of study participants (eg demographic, clinical, social) and information on exposures and potential confounders |  | 10 |
|  |  | (b) Indicate number of participants with missing data for each variable of interest |  | Figure 1 |
|  |  | (c) Summarise follow-up time (eg, average and total amount) |  | Table 1 |
| Outcome data | 15* | Report numbers of outcome events or summary measures over time |  | Figures  2 & 3 |
| Main results | 16 | (*a*) Give unadjusted estimates and, if applicable, confounder-adjusted estimates and their precision (eg, 95% confidence interval). Make clear which confounders were adjusted for and why they were included |  | Figure 2,  11-12 |
|  |  | (*b*) Report category boundaries when continuous variables were categorized |  | n/a |
|  |  | (*c*) If relevant, consider translating estimates of relative risk into absolute risk for a meaningful time period |  |  |
| Other analyses | 17 | Report other analyses done—eg analyses of subgroups and interactions, and sensitivity analyses |  | 12-13 |
| **Discussion** | | |  |  |
| Key results | 18 | Summarise key results with reference to study objectives |  | 13-14 |
| Limitations | 19 | Discuss limitations of the study, taking into account sources of potential bias or imprecision. Discuss both direction and magnitude of any potential bias |  | 16-17 |
| Interpretation | 20 | Give a cautious overall interpretation of results considering objectives, limitations, multiplicity of analyses, results from similar studies, and other relevant evidence |  | 13-17 |
| Generalisability | 21 | Discuss the generalisability (external validity) of the study results |  | 15, 17 |
| **Other information** | | |  |  |
| Funding | 22 | Give the source of funding and the role of the funders for the present study and, if applicable, for the original study on which the present article is based |  | n/a |

*Give information separately for exposed and unexposed groups.

**Note:** An Explanation and Elaboration article discusses each checklist item and gives methodological background and published examples of transparent reporting. The STROBE checklist is best used in conjunction with this article (freely available on the Web sites of PLoS Medicine at http://www.plosmedicine.org/, Annals of Internal Medicine at http://www.annals.org/, and Epidemiology at http://www.epidem.com/). Information on the STROBE Initiative is available at <http://www.strobe-statement.org>.

**eTable 2.** Diagnostic Codes Used in the Study with Relevant References

1. EXPOSURE

Major congenital anomalies by organ system (based on 2014 EUROCAT categorization of subgroups of congenital anomalies)

|  | **ICD 8** | **ICD 10** |
| --- | --- | --- |
| All congenital anomalies | 740-759, *excluding* the | All Q chapter *excluding* the |
|  | following minor | following minor congenital |
|  | congenital anomalies | anomalies |
|  | Exclusions: 745.19- | Exclusions: Q671, Q674, |
|  | 745.99, 747.09 (if | Q672, Q189, Q670, Q673, |
|  | gestational age < 37 | Q753, Q135, Q101, Q102, |
|  | weeks and no other | Q752, Q103, Q105,Q170, |
|  | congenital heart | Q171, Q172, Q173, Q174, |
|  | disease), 750.09, | Q175, Q179, Q180, Q181, |
|  | 750.19, 751.09, 752.10 | Q182, Q189, Q385, Q186, |
|  | - 752.19, 752.84, | Q382, Q184, Q187, Q185, |
|  | 755.69, 756.81, | Q674, Q381, Q189, Q680, |
|  | 756.29, 756.30, 756.31, | Q740G, Q845, Q8280, Q653- |
|  | 765.39, 757.10, 757.11, | 656, Q668, Q669, Q665, |
|  | 757.12, 757.13, 757.18 | Q845, Q663, Q666, Q662, |
|  |  | Q667, Q664, Q833, Q825, |
|  | Also include: 761.39 | Q766, Q765, Q683, Q684, |
|  | (congenital rubella) and | Q685, Q675, Q764L, Q676, |
|  | 761.49 (congenital | Q682A, Q677, Q678, Q760, |
|  | toxoplasmosis) | Q767C, Q270, Q250 (if GA < |
|  |  | 37 weeks), Q211C, Q256 (if |
|  |  | GA < 37 weeks), Q261, |
|  |  | Q254E, Q331, Q314, Q315, |
|  |  | Q320, Q401, Q430, Q400, |
|  |  | Q633, Q610, Q627, Q552F, |
|  |  | Q527, Q525, Q523, Q552, |
|  |  | Q53, Q899, Q950, Q951 |
|  |  | Q53 and Q65 specifically |
|  |  | excluded due to poor validity |
|  |  | in Danish National Patient |
|  |  | Register |
|  |  | Also include (add) D215 |
|  |  | (benign neoplasm of pelvis), |
|  |  | D821 (DiGeorge Syndrome), |
|  |  | D1810 (lymphangioma), |
|  |  | P350, P351, P371 |
| NERVOUS SYSTEM | 740-743.99 | Q00-Q07 |

|  | **ICD 8** | **ICD 10** |
| --- | --- | --- |
| EYE | 744-744.99 | Q10-Q15 (exclude Q101-  Q103, Q105, Q135) |
| EAR, FACE and NECK | 745.0-745.09, (exclude  745.19-745.99, 756.81) | Q16-Q18 (exclude Q170- Q175, Q179, Q180-Q182,  Q184-Q187, Q189) |
| CONGENITAL HEART DISEASE | 746, 747 (for 747.09  patent ductus arteriosus, exclude if gestational age < 37 weeks and no other congenital heart disease) | Q20-Q26 (exclude Q250 (if GA < 37 weeks), Q211C,  Q256 (if GA < 37 weeks), Q254E, Q261 |
| RESPIRATORY | 748 | Q30-34 (exclude Q314, Q315,  Q320, Q331) |
| ORO-FACIAL CLEFT | 749 | Q35-Q37 |
| DIGESTIVE SYSTEM | 750.2-750.99 [exclude  750.09 (tongue anomalies) and 750.19 (pyloric stenosis)], 756.80 | Q38-Q45, Q790 (exclude Q381, Q382, Q385, Q400, Q401, Q430) |
| ABDOMINAL WALL DEFECTS | 751.1–751.99 [exclude  751.09, Meckel’s diverticulum] | Q792, Q793, Q795 |
| URINARY | 752 (exclude 752.10-  752.19) | Q60-Q64, Q794 (exclude  Q610, Q627, Q633) |
| OTHER EXCLUSIONS |  | Q760, Q764L, Q765, Q766, Q767C, Q825, Q8280, Q833,  Q899, Q950, Q951, Q845 |
| OTHER  ANOMALIES/SYNDROMES | As below | As below |
| Skeletal Dysplasia | 756.48, 756.49, 756.59,  756.60, 756.4 | Q740B, Q77, Q780, Q782-  Q788 |
| Craniosynostosis | 756.00 | Q750 |
| Congenital constriction  bands/amniotic bands | No code | No code |
| Situs Inversus | 759.00-759.09 | Q893 |
| Conjoined Twins | 759.19 | Q894 |
| Congenital skin disorders | 757.20-757.99 | Q80-Q82 |
| VATER/VACTERL | No code | Q872G |
| Vascular Disruption Anomalies | 751.11, 751.19,  755.24-755.25, 755.49,  755.28, 755.31, 751.82,  756.89 | Q411, Q412, Q418, Q710, Q712, Q713, Q720, Q722, Q723, Q730, Q793, Q795,  Q798S, Q870G |
| Laterality Anomalies | 745.89, 748.69, 759.00,  759.01, 759.09 | Q206, Q240, Q890, Q893 |

|  | **ICD 8** | **ICD 10** |
| --- | --- | --- |
| Teratogenic syndromes with  malformations | 761.39 | Q86, P350, P351, P371 |
| Fetal Alcohol Syndrome | No code | Q860 |
| Valproate syndrome | No code | No code |
| Maternal infections resulting  in malformations | 761.39 | P350, P351, P371 |
| Genetic Syndromes and  Microdeletions | 753.10 | Q447B, Q619A, Q751, Q754,  Q87, Q936, D821 |
| Chromosomal | 759 | Q90-Q92; Q93, Q96-99\ |

Note: At least 1 code in two or more organ systems required for inclusion as multi-organ major congenital anomalies.

2. COVARIATES

(1) Charlson Comorbidity Index

| Conditions and Weight | | **ICD 8** | **ICD 10** |
| --- | --- | --- | --- |
| Myocardial infarction | Weight 1 | 410 | I21;I22;I23 |
| Congestive heart  failure | Weight 1 | 427.09; 427.10; 427.11;  427.19; 428.99; 782.49 | I50; I11.0; I13.0; I13.2 |
| Peripheral vascular  disease | Weight 1 | 440; 441; 442; 443;  444; 445 | I70; I71; I72; I73; I74;  I77 |
| Cerebrovascular  disease | Weight 1 | 430-438 | I60-I69; G45; G46 |
| Dementia | Weight 1 | 290.09-290.19; 293.09 | F00-F03; F05.1; G30 |
| Chronic pulmonary disease | Weight 1 | 490-493; 515-518 | J40-J47; J60-J67; J68.4; J70.1; J70.3;  J84.1; J92.0; J96.1; J98.2; J98.3 |
| Connective tissue disease | Weight 1 | 712; 716; 734; 446;  135.99 | M05; M06; M08; M09; M30; M31; M32; M33;  M34; M35; M36; D86 |
| Ulcer disease | Weight 1 | 530.91; 530.98; 531-  534 | K22.1; K25-K28 |
| Mild liver disease | Weight 1 | 571; 573.01; 573.04 | B18; K70.9; K71; K73;  K74; K76.0 |
| Diabetes without end- organ damage | Weight 1 | 249.00; 249.06; 249.07;  249.09; 250.00; 250.06;  250.07; 250.09 | E10.0, E10.1; E10.9; E11.0; E11.1; E11.9 |
| Diabetes with end-  organ damage | Weight 2 | 249.01-249.05; 249.08;  250.01-250.05; 250.08 | E10.2-E10.8, E11.2-  E11.8 |
| Hemiplegia | Weight 2 | 344 | G81; G82 |
| Moderate to severe renal disease | Weight 2 | 403; 404; 580-583; 584;  590.09; 593.19; 753.10-  753.19; 792 | I12; I13; N00-N05; N07; N11; N14; N17-  N19; Q61 |
| Non-metastatic solid  tumour | Weight 2 | 140-194 | C00-C75 |
| Leukaemia | Weight 2 | 204-207 | C91-C95 |
| Lymphoma | Weight 2 | 200-203; 275.59 | C81-C85; C88; C90;  C96 |
| Moderate to severe liver disease | Weight 3 | 070.00; 070.02; 070.04; 070.06; 070.08; 573.00;  456.00–456.09 | B15.0; B16.0; B16.2; B19.0; K70.4; K72; K76.6; I85 |
| Metastatic cancer | Weight 6 | 195–198; 199 | C76–C80 |
| AIDS | Weight 2 | 079.83 | B21–B24 |

Note: ICD-10 codes K70.0-K70.3 (mild liver disease) and K70.4 (moderate to severe liver disease) codes removed as these are liver diseases associated with alcohol-related disease.

(2) Spontaneous Abortion

|  | **ICD8** | **ICD10** |
| --- | --- | --- |
| Missed Abortion | 6346x | O021, O021A |
| Missed Abortion with dilation and  curettage | 6451x |  |
| Spontaneous Abortion |  | O03x |
| Spontaneous Abortion, other | 6438x |  |
| Spontaenous Abortion with  complications | 6439x |  |

(3) Pregnancy complications

|  | **ICD 8** | **ICD 10** |
| --- | --- | --- |
| A) Placental |  |  |
| Pre-eclampsia | 63703, 63704, 63709 | O140—O142, O149, O150-  O159 |
| Gestational or unspecified  Hypertension | 63700 | O13, O16 |
| Placental abruption | 63219, 6515 | O45 |
| Placental infarction | No code | O43.1, O43.8,  O43.9 |
|  |  |  |
| B) Non-Placental |  |  |
| Intrauterine hypoxia and birth  asphyxia | 77640–77650, 76840, 76890 | P20, P21 |
| Uterine rupture | 659 | O71.0, O71.1 |
| Umbilical cord prolapse or vasa  previa | 663.3, 663.4 | O69 |
| Amniotic fluid embolism | 673.1 | O88.1 |
| Fetal-maternal hemorrhage | No code | O43.0 |
| Chorioamnionitis | No code | O41.1 |

Other notes on covariates:

Marital status was ascertained from Danish Civil Registry at the time of the index birth. A six-month allowance was made post-index birth to allow for delayed registration of marriage.

3. OTHER HEALTH CONDITIONS

(1) Psychiatric disorder

| **ICD 8** | **ICD 10** |
| --- | --- |
| 291-319 | F04- F99 |
| **Anatomical Therapeutic Chemical Classification System (ATC) codes** | |
| N05A, excluding N05AN01 (lithium), N06A (depression) | |

(2) Diabetes defined using prescription drugs

| **Anatomical Therapeutic Chemical Classification System (ATC) codes** |
| --- |
| A10A and A10B |

**eTable 3.** List of Dream Codes Used to Measure Employment Status

| **Codes**  **(Current)** | **Codes**  **(Past)** | **Description** | **Employment**  **Status** |
| --- | --- | --- | --- |
| 521 |  | Trainee, adult | Outside workforce |
| 413 |  | Leave-of-absence due to education | Outside workforce |
| 651, 652, 661 | 662, 794 | State Education Fund grants | Outside workforce |
| No entry | No entry | No transfer payment | Employed |
|  | 795 | Benefits due to sick child | Outside workforce |
| 115 | 112, 113 | Unemployment benefit part time | Unemployed |
|  | 122, 123 | Vacation payment from employment | Employed |
| 412 | 411 | Leave-of-absence schemes | Outside workforce |
| 121 |  | Vacation payment | Unemployed |
| 881 |  | Maternity leave pay | Outside workforce |
| 111 |  | Unemployment benefit all week | Unemployed |
|  | 114 | Unemployed without benefit | Unemployed |
|  | 124-126 | Vacation payment from unemployment | Unemployed |
| 130, 133  134-139  152, 153  730, 732-739 | 131,132,  141, 142, 731, 741 | Social assistance, not health related | Outside workforce |
| 160  163-169 |  | Ready for employment benefit for immigrants | Outside workforce |
| 710  713-719 | 711,712 | Social benefit, immigrants | Outside workforce |
| 704-709 |  | Immigration benefit during special efforts e.g. job training | Outside workforce |
| 140, 143-149, 151, 700, 703, 720  723-729 | 141,142  414,722, 721, 732, 742, 751, 752 | Education assistance, not health related | Outside workforce |
| 213-219  231,299  511,522  722 | 211, 212  221, 222, 224, 225  232, 297, 298, 541, 759 | Unemployment benefit during special efforts e.g. job training or supervision. | Unemployed |
| 771, 774, 895 | 761,762, 769,772, 773,779, 782,796, | Flexible job for those with a reduced workability | Outside workforce |
| 774, 890, 893-899 | 891, 892 | Sick leave benefit | Outside workforce |
| 740  743-748 |  | Unemployed awaiting flexible job | Outside workforce |
| 750, 753-758, 760  763-768 | 791, 792 | Rehabilitation | Outside workforce |
| 810  813-819 | 784 | Vocational rehabilitation program | Outside workforce |
| 870  873-879 | 785 | Workability clarification | Outside workforce |
| 781, 783 | 797, 793 | Disability pension |  |

**eTable 4.** Stratified Analysis: Rate of Being Outside Workforce in the Mothers of Children with Major Congenital Anomalies Cohort by Pre-Pregnancy Psychiatric Medical History compared to the Matched Comparison Cohort

|  | Sample Size | |  |
| --- | --- | --- | --- |
|  | Exposed Cohort (MCMCA) | Comparison Cohort  (Non-MCMCA) | Adjusted Rate Ratio (95% CI) |
| Outside Workforce |  |  |  |
| First year after delivery |  |  |  |
| No Pre-Pregnancy Psychiatric History | 19,518 | 199,033 | 0.98 (0.98–0.99) |
| Pre-Pregnancy Psychiatric History | 4,119 | 35,550 | 0.99 (0.99–1.00) |
| 1-6 years after delivery |  |  |  |
| No Pre-Pregnancy Psychiatric History | 19,458 | 198,308 | 1.05 (1.04–1.07) |
| Pre-Pregnancy Psychiatric History | 4,110 | 35,455 | 1.02 (0.99–1.05) |
| 7-13 years after delivery |  |  |  |
| No Pre-Pregnancy Psychiatric History | 15,042 | 152,513 | 1.09 (1.06–1.13) |
| Pre-Pregnancy Psychiatric History | 2,554 | 21,948 | 1.04 (0.99–1.10) |
| 14-18 years after delivery |  |  |  |
| No Pre-Pregnancy Psychiatric History | 8,962 | 90,169 | 1.14 (1.08–1.20) |
| Pre-Pregnancy Psychiatric History | 926 | 8,054 | 1.03 (0.91–1.15) |

Note: Adjusted covariates included maternal demographics (age at delivery, marital status, income quartile, level of education ascertained as of the year prior to the index birth, and immigration status), pregnancy history (parity and pregnancy-related health conditions), health (Charlson Comorbidity Index score), and infant’s year of birth.

**eTable 5.** Stratified Analysis: Rate of Being Outside Workforce in the Mothers of Children with Major Congenital Anomalies Cohort by Pregnancy-related Complications Index compared to the Matched Comparison Cohort

|  | Sample Size | |  |
| --- | --- | --- | --- |
|  | Exposed Cohort (MCMCA) | Comparison Cohort  (Non-MCMCA) | Adjusted Rate Ratio (95% CI) |
| Outside Workforce |  |  |  |
| First year after delivery |  |  |  |
| No Complications | 21,813 | 222,764 | 0.99 (0.98–0.99) |
| Pregnancy-related Complications | 1824 | 11,819 | 0.98 (0.97–1.00) |
| 1-6 years after delivery |  |  |  |
| No Complications | 21,750 | 221,989 | 1.05 (1.04–1.07) |
| Pregnancy-related Complications | 1818 | 11,774 | 0.99 (0.95–1.04) |
| 7-13 years after delivery |  |  |  |
| No Complications | 16,286 | 166,014 | 1.10 (1.07–1.13) |
| Pregnancy-related Complications | 1310 | 8447 | 0.99 (0.90–1.08) |
| 14-18 years after delivery |  |  |  |
| No Complications | 9209 | 93,753 | 1.13 (1.07–1.19) |
| Pregnancy-related Complications | 679 | 4470 | 1.06 (0.89–1.26) |

Note: Adjusted covariates included maternal demographics (age at delivery, marital status, income quartile, level of education ascertained as of the year prior to the index birth, and immigration status), pregnancy history (parity), health (Charlson Comorbidity Index score, pre-birth mental illness), and infant’s year of birth.

**eTable 6.** Stratified Analysis: Rate of Being Outside Workforce in the Mothers of Children with Major Congenital Anomalies Cohort by Charlson Comorbidity Index (CCI) compared to the Matched Comparison Cohort

|  | Sample Size | |  |
| --- | --- | --- | --- |
|  | Exposed Cohort (MCMCA) | Comparison Cohort  (Non-MCMCA) | Adjusted Rate Ratio (95% CI) |
| Outside Workforce |  |  |  |
| First year after delivery |  |  |  |
| CCI Score 0 | 21,730 | 219,302 | 0.98 (0.98–0.99) |
| CCI Score 1 | 1440 | 12,174 | 0.99 (0.98–1.01) |
| CCI Score ≥ 2 | 467 | 3107 | 1.02 (1.00–1.05) |
| 1-6 years after delivery |  |  |  |
| CCI Score 0 | 21,665 | 218,541 | 1.05 (1.03–1.07) |
| CCI Score 1 | 1438 | 12,130 | 1.05 (1.00–1.11) |
| CCI Score ≥ 2 | 465 | 3092 | 1.06 (0.96–1.18) |
| 7-13 years after delivery |  |  |  |
| CCI Score 0 | 16,348 | 164,565 | 1.09 (1.06–1.12) |
| CCI Score 1 | 941 | 7881 | 1.09 (0.99–1.20) |
| CCI Score ≥ 2 | 307 | 2015 | 1.21 (1.00–1.45) |
| 14-18 years after delivery |  |  |  |
| CCI Score 0 | 9356 | 93,942 | 1.13 (1.08–1.19) |
| CCI Score 1 | 417 | 3368 | 1.05 (0.85–1.30) |
| CCI Score ≥ 2 | 115 | 913 | 1.04 (0.74–1.47) |

Note: Adjusted covariates included maternal demographics (age at delivery, marital status, income quartile, level of education ascertained as of the year prior to the index birth, and immigration status), pregnancy history (parity and pregnancy-related health conditions), health (pre-birth mental illness), and infant’s year of birth.

**eTable 7.** Stratified Analysis: Rate of Receiving Disability Pension in the Mothers of Children with Major Congenital Anomalies Cohort by Pre-Pregnancy Psychiatric Medical History compared to the Matched Comparison Cohort

| Pre-Pregnancy Psychiatric History | Incidence Rates (95% CI)  per 1000 years | | Adjusted  Hazard Ratio  (95% CI) |
| --- | --- | --- | --- |
|  | MCMCA  Cohort | Comparison  Cohort |  |
| No Pre-Pregnancy Psychiatric History | 2.53 (2.32 - 2.73) | 1.89 (1.83 - 1.94) | 1.24 (1.13 - 1.35) |
| Pre-Pregnancy Psychiatric History | 6.96 (6.07 - 7.84) | 5.90 (5.62 - 6.18) | 1.11 (0.96 - 1.27) |

**eTable 8.** Stratified Analysis: Rate of Receiving Disability Pension in the Mothers of Children with Major Congenital Anomalies Cohort by Charlson Comorbidity Index (CCI) compared to the Matched Comparison Cohort

| CCI Score | Incidence Rates (95% CI) per 1000 years | | Adjusted Hazard Ratio (95% CI) |
| --- | --- | --- | --- |
|  | MCMCA Cohort | Comparison Cohort |  |
| CCI score 0 | 2.85 (2.63 - 3.06) | 2.22 (2.16 - 2.28) | 1.18 (1.09 - 1.28) |
| CCI score 1 | 6.10 (4.76 - 7.44) | 4.38 (3.99 - 4.77) | 1.27 (1.00 - 1.61) |
| CCI score 2+ | 8.69 (5.85 - 11.53) | 5.19 (4.35 - 6.03) | 1.54 (1.07 - 2.23) |

**eTable 9.** Stratified Analysis: Rate of Being Outside Workforce in the Mothers of Children with Major Congenital Anomalies Cohort by Education compared to the Matched Comparison Cohort

|  | Sample Size | |  |
| --- | --- | --- | --- |
|  | Exposed Cohort (MCMCA) | Comparison Cohort  (Non-MCMCA) | Adjusted Rate Ratio (95% CI) |
| Outside Workforce |  |  |  |
| First year after delivery |  |  |  |
| Education, Primary/Secondary | 8088 | 76,113 | 0.99 (0.99–1.00) |
| Education, Vocational/Post-Secondary | 7675 | 74,937 | 0.98 (0.97–0.99) |
| Education, Bachelor’s or higher | 6952 | 74,610 | 0.98 (0.97–0.99) |
| 1-6 years after delivery |  |  |  |
| Education, Primary/Secondary | 8070 | 75,907 | 1.06 (1.04–1.07) |
| Education, Vocational/Post-Secondary | 7664 | 74,804 | 1.04 (1.01–1.07) |
| Education, Bachelor’s or higher | 6938 | 74,406 | inestimable |
| 7-13 years after delivery |  |  |  |
| Education, Primary/Secondary | 6335 | 60,090 | 1.08 (1.05–1.12) |
| Education, Vocational/Post-Secondary | 6019 | 59,103 | 1.07 (1.02–1.12) |
| Education, Bachelor’s or higher | 4833 | 51,255 | 1.12 (1.06–1.19) |
| 14-18 years after delivery |  |  |  |
| Education, Primary/Secondary | 3836 | 36,438 | 1.11 (1.05–1.17) |
| Education, Vocational/Post-Secondary | 3563 | 35,694 | 1.14 (1.05–1.23) |
| Education, Bachelor’s or higher | 2299 | 24,379 | 1.15 (1.01–1.31) |

Note: Adjusted covariates included maternal demographics (age at delivery, marital status, income quartile as of the year prior to the index birth, and immigration status), pregnancy history (parity and pregnancy-related health conditions), health (Charlson Comorbidity Index score, pre-birth mental illness), and infant’s year of birth.

**eTable 10.** Stratified Analysis: Rate of Being Employed in the Mothers of Children with Major Congenital Anomalies Cohort by the year of delivery compared to the Matched Comparison Cohort

|  | Sample Size | |  | |
| --- | --- | --- | --- | --- |
|  | Exposed Cohort (MCMCA) | Comparison Cohort  (Non-MCMCA) | Adjusted Rate Ratio (95% CI) |  |
| 1997 | 1130 | 11,225 | 1.26 (1.18–1.35) |  |
| 1998 | 1100 | 10,915 | 1.26 (1.18–1.36) |  |
| 1999 | 1099 | 10,906 | 1.28 (1.19–1.37) |  |
| 2000 | 1123 | 11,158 | 1.29 (1.21–1.38) |  |
| 2001 | 1078 | 10,715 | 1.23 (1.14–1.33) |  |
| 2002 | 1123 | 11,162 | 1.10 (1.00–1.22) |  |
| 2003 | 1145 | 11,378 | 1.14 (1.04–1.25) |  |
| 2004 | 1159 | 11,528 | 1.15 (1.04–1.27) |  |
| 2005 | 1160 | 11,538 | 1.15 (1.05–1.26) |  |
| 2006 | 1175 | 11,669 | 1.01 (0.92–1.10) |  |
| 2007 | 1087 | 10,790 | 1.05 (0.96–1.14) |  |
| 2008 | 1031 | 10,220 | 1.05 (0.95–1.15) |  |
| 2009 | 1089 | 10,778 | 1.04 (0.95–1.13) |  |
| 2010 | 1167 | 11,565 | 0.92 (0.84–0.99) |  |
| 2011 | 1013 | 10,046 | 1.00 (0.91–1.11) |  |
| 2012 | 1163 | 11,517 | 0.91 (0.83–1.00) |  |
| 2013 | 1171 | 11,579 | 1.02 (0.93–1.11) |  |
| 2014 | 1177 | 11,662 | 0.95 (0.87–1.04) |  |
| 2015 | 1165 | 11,569 | 1.06 (0.97–1.17) |  |
| 2016 | 1181 | 11,722 | 0.97 (0.89–1.06) |  |
| 2017 | 1101 | 10,941 | 0.97 (0.89–1.06) |  |

Note: Adjusted covariates included maternal demographics (age at delivery, marital status, income quartile, level of education ascertained as of the year prior to the index birth, and immigration status), pregnancy history (parity and pregnancy-related health conditions) and health (Charlson Comorbidity Index score, pre-birth mental illness).

**eTable 11.** Stratified Analysis: Rate of Being Employed in the Mothers of Children with Major Congenital Anomalies Cohort by the year of delivery compared to the Matched Comparison Cohort

|  | Sample Size | |  |
| --- | --- | --- | --- |
|  | Exposed Cohort (MCMCA) | Comparison Cohort  (Non-MCMCA) | Adjusted Rate Ratio (95% CI) |
| Outside Workforce |  |  |  |
| First year after delivery |  |  |  |
| Single-organ MCA | 21,101 | 209,448 | 0.99 (0.99–0.99) |
| Multiple-organ MCAs | 2536 | 25,135 | 0.94 (0.93–0.95) |
| 1-6 years after delivery |  |  |  |
| Single-organ MCA | 21,039 | 208,726 | 1.05 (1.03–1.06) |
| Multiple-organ MCAs | 2529 | 25,037 | 1.08 (1.04–1.13) |
| 7-13 years after delivery |  |  |  |
| Single-organ MCA | 15,626 | 155,015 | 1.08 (1.06–1.11) |
| Multiple-organ MCAs | 1970 | 19,446 | 1.13 (1.05–1.21) |
| 14-18 years after delivery |  |  |  |
| Single-organ MCA | 8676 | 86,148 | 1.12 (1.06–1.18) |
| Multiple-organ MCAs | 1212 | 12,075 | 1.18 (1.02–1.36) |

Note:

1. Adjusted covariates included maternal demographics (age at delivery, marital status, income quartile, level of education ascertained as of the year prior to the index birth, and immigration status), pregnancy history (parity and pregnancy-related health conditions), health (Charlson Comorbidity Index score, pre-birth mental illness), and infant’s year of birth.

2. Single-organ MCAs and multi-organ MCAs were compared to their matched comparison cohorts.

**eTable 12.** Sensitivity Analysis: Examining employment status using various thresholds of labor market participation, “employed” referred to those who participated in the labor market during at least 50%, 75%, or 90% of the given period.

| Work participation score >50% (i.e., employed) | Adjusted Risk Ratio (95% CI) |
| --- | --- |
| First year after delivery | 1.17 (1.13–1.22) |
| 1-6 years after delivery | 0.98 (0.97–0.99) |
| 7-13 years after delivery | 0.97 (0.96–0.98) |
| 14-18 years after delivery | 0.95 (0.94–0.97) |
| Work participation score >75% (i.e., employed) | Adjusted Risk Ratio (95% CI) |
| First year after delivery | 1.10 (1.01–1.19) |
| 1-6 years after delivery | 0.97 (0.96–0.99) |
| 7-13 years after delivery | 0.97 (0.96–0.98) |
| 14-18 years after delivery | 0.96 (0.94–0.97) |
| Work participation score >90% (i.e., employed) | Adjusted Risk Ratio (95% CI) |
| First year after delivery | 1.02 (0.93–1.11) |
| 1-6 years after delivery | 0.99 (0.97–1.02) |
| 7-13 years after delivery | 0.96 (0.94–0.98) |
| 14-18 years after delivery | 0.95 (0.93–0.98) |

Note: Adjusted covariates included maternal demographics (age at delivery, marital status, income quartile, level of education ascertained as of the year prior to the index birth, and immigration status), pregnancy history (parity and pregnancy-related health conditions), health (Charlson Comorbidity Index score, pre-birth mental illness), and infant’s year of birth.

**eTable 13.** Sensitivity Analysis: Rate of Being Outside Workforce in the Mothers of Children with Major Congenital Anomalies Cohort compared to the Matched Comparison Cohort, excluding mothers not attached to labor market prior to delivery.

| Outside Workforce | Adjusted Rate Ratio (95% CI) |
| --- | --- |
| First year after delivery | 0.98 (0.97–0.98) |
| 1-6 years after delivery | 1.03 (1.00–1.05) |
| 7-13 years after delivery | 1.06 (1.02–1.10) |
| 14-18 years after delivery | 1.12 (1.04–1.21) |

Note:

(1) Attachment to labor market was defined as labor market participation at least 75% one year before beginning maternity leave for those who received the maternity leave benefit or one year before the delivery date for mothers who did not receive the maternity leave benefit

(2) Adjusted covariates included maternal demographics (age at delivery, marital status, income quartile, level of education ascertained as of the year prior to the index birth, and immigration status), pregnancy history (parity and pregnancy-related health conditions), health (Charlson Comorbidity Index score, pre-birth mental illness), and infant’s year of birth.

**eTable 14.** Sensitivity Analysis: Employment Status of Mothers with At Least 75% Labor Market Participation, Excluding Non-Active Labor Market Participation Prior to Delivery.

| Work participation score >75% (i.e., employed) | Adjusted Risk Ratio (95% CI) |
| --- | --- |
| First year after delivery | 1.11 (1.02–1.20) |
| 1-6 years after delivery | 0.98 (0.96–1.00) |
| 7-13 years after delivery | 0.99 (0.97–1.00) |
| 14-18 years after delivery | 0.98 (0.96–1.00) |

Note:

(1) Attachment to labor market defined as labor market participation at least 75% one year before beginning maternity leave for those who received the maternity leave benefit or one year before the delivery date for mothers who did not receive the maternity leave benefit

(2) Adjusted covariates included maternal demographics (age at delivery, marital status, income quartile, level of education ascertained as of the year prior to the index birth, and immigration status), pregnancy history (parity and pregnancy-related health conditions), health (Charlson Comorbidity Index score, pre-birth mental illness), and infant’s year of birth.

**eTable 15.** Sensitivity Analysis: The Mothers of Children with Major Congenital Anomalies Cohort vs. the Matched Comparison Cohort for Body Mass Index in the 2004-2017 period

|  | Adjusted Rate Ratio (95% CI) |
| --- | --- |
| Employed |  |
| First year after delivery | 1.02 (0.99-1.04) |
| 1-6 years after delivery | 0.98 (0.97-0.99) |
| 7-13 years after delivery | 0.97 (0.96-0.99) |
| 14-18 years after delivery | 0.97 (0.93-1.00) |
| Outside Workforce |  |
| First year after delivery | 1.00 (1.00-1.00) |
| 1-6 years after delivery | 1.04 (1.03-1.06) |
| 7-13 years after delivery | 1.07 (1.04-1.11) |
| 14-18 years after delivery | 1.12 (0.99-1.26) |
| Unemployed |  |
| First year after delivery | 0.77 (0.71-0.82) |
| 1-6 years after delivery | 0.94 (0.90-0.98) |
| 7-13 years after delivery | 1.01 (0.95-1.08) |
| 14-18 years after delivery | 1.02 (0.81-1.29) |

Note: Adjusted covariates included maternal demographics (age at delivery, marital status, income quartile, level of education ascertained as of the year prior to the index birth, and immigration status), pregnancy history (parity and pregnancy-related health conditions), health (Charlson Comorbidity Index score, pre-birth mental illness), infant’s year of birth and body mass index.

**eTable 16.** Sensitivity Analysis: Association between MCMCAs and Employment Status, Stratified by Year 2013

|  | Adjusted Rate Ratio (95% CI) | |  |
| --- | --- | --- | --- |
| Outcomes | 1997-2012 | 2013-2017 |  |
| Employed |  |  |  |
| First year after delivery | 1.13 (1.11–1.16) | 0.99 (0.96–1.03) |  |
| 1-6 years after delivery | 0.98 (0.97–0.99) | 0.99 (0.97–1.01) |  |
| 7-13 years after delivery | 0.97 (0.96–0.98) |  |  |
| 14-18 years after delivery | 0.96 (0.94–0.97) |  |  |
| Outside Workforce |  |  |  |
| First year after delivery | 0.98 (0.97–0.98) | 1.01 (1.00–1.01) |  |
| 1-6 years after delivery | 1.05 (1.04–1.07) | 1.03 (1.00–1.06) |  |
| 7-13 years after delivery | 1.10 (1.07–1.13) |  |  |
| 14-18 years after delivery | 1.12 (1.07–1.18) |  |  |
| Unemployed |  |  |  |
| First year after delivery | 0.76 (0.71–0.81) | 0.76 (0.68–0.84) |  |
| 1-6 years after delivery | 0.94 (0.91–0.97) | 0.93 (0.87–1.00) |  |
| 7-13 years after delivery | 0.97 (0.93–1.01) |  |  |
| 14-18 years after delivery | 0.97 (0.90–1.05) |  |  |

Note: Adjusted covariates included maternal demographics (age at delivery, marital status, income quartile, level of education ascertained as of the year prior to the index birth, and immigration status), pregnancy history (parity and pregnancy-related health conditions), health (Charlson Comorbidity Index score, pre-birth mental illness), and infant’s year of birth.

**eTable 17.**  Sensitivity Analysis: Association between being MCMCA and Receiving Disability Pension

1) Defining vocational training and flexible job as disability pension, effective January 1, 2013

| Incidence Rates (95% CI) per 1000 years | | Adjusted Hazard Ratio (95% CI) |
| --- | --- | --- |
| MCMCA Cohort | Comparison Cohort |  |
| 7.97 (7.62 - 8.31) | 6.37 (6.28 - 6.47) | 1.16 (1.11 - 1.21) |

2) Defining vocational training and flexible job as disability pension throughout the entire study period

| Incidence Rates (95% CI) per 1000 years | | Adjusted Hazard Ratio (95% CI) |
| --- | --- | --- |
| MCMCA Cohort | Comparison Cohort |  |
| 7.68 (7.34 - 8.02) | 6.21 (6.11 - 6.31) | 1.15 (1.09 - 1.20) |

3) Censoring follow-up time on December 31, 2012 for those who gave birth before the change in the disability pension policy and stratifying by year 2013

| Adjusted Hazard Ratio (95% CI) | |
| --- | --- |
| 1997-2012 | 2013-2017 |
| 1.21 (1.10 - 1.32) | 1.21 (0.67 - 2.19) |

Note: Adjusted covariates included maternal demographics (age at delivery, marital status, income quartile, level of education ascertained as of the year prior to the index birth, and immigration status), pregnancy history (parity and pregnancy-related health conditions), health (Charlson Comorbidity Index score, pre-birth mental illness), and infant’s year of birth.

**eFigure 1.** Annual Mean Number of Weeks in Employment

Abbreviation: MCMCA: mothers of children with major congenital anomalies

**eFigure 2.** Annual Mean Number of Weeks in Outside Workforce

Abbreviation: MCMCA: mothers of children with major congenital anomalies

**eFigure 3.** Annual Mean Number of Weeks in Unemployment

Abbreviation: MCMCA: mothers of children with major congenital anomalies
